# ﻿A new species of *Nitocra* Boeck, 1865 (Copepoda, Harpacticoida) from Vietnam

**DOI:** 10.3897/zookeys.1258.167766

**Published:** 2025-11-06

**Authors:** Ngoc-Son Tran, Thi-Phuong Pham, Khanh Chuyen Phung, Samuel Gómez

**Affiliations:** 1 The University of Danang – University of Science and Education, 459 Ton Duc Thang St., Danang City 550000, Vietnam The University of Danang – University of Science and Education Danang Vietnam; 2 Universidad Nacional Autónoma de México, Instituto de Ciencias del Mar y Limnología, Unidad Académica Mazatlán, Joel Montes Camarena s/n, Mazatlán, 82040, Sinaloa, Mexico Universidad Nacional Autónoma de México Mazatlán Mexico

**Keywords:** Groundwater fauna, hyporheic zone, new species, *
Nitokra
*, Southeast Asia, species groups, taxonomy

## Abstract

An undescribed taxon of the species-rich genus *Nitocra* was found during a survey on the harpacticoid copepods from the hyporheic zone in Vu Gia–Thu Bon Rriver, Central Vietnam. The new species, *N. duyxuyenensis***sp. nov.**, is morphologically similar to the American *N. evergladensis* Bruno & Reid, 2002. These two species can be separated by the number of marginal spinules on the anal operculum, the relative length of the baseoendopod of the female fifth leg, the structure of the antenna, the number of setae on the female fifth leg, the number of setae on the inner margin of the first endopodal segment of the second to fourth swimming legs of the males, and the number of setae on the third endopodal segment of the third swimming leg. Additionally, some comments on the groups and subgroups of the genus defined so far are given, with the proposal of the *lacustris* species subgroup for group III of Gómez et al. (2012).

## ﻿Introduction

With 74 species and 26 subspecies, the genus *Nitocra* Boeck, 1865 is one of the genera with the largest number of species in the family Ameiridae. Of these, nine species (*N. arctolongus* Shen & Tai, 1973; *N. karanovici* Chullasorn, Kangtia & Klangsin, 2014; *N. koreanus*Chang 2007; *N. malaica* Kiefer, 1929; *N. platypus platypus* Daday, 1906; *N. platypus bakeri* Chappuis, 1930; *N. sewelli* Gurney, 1927; *N. vietnamensis* Tran & Chang, 2012; *N. quangnamensis* Tran, Trinh-Dang, Nguyen & Brancelj, 2025) have been found in Asia. Species of *Nitocra* are common in several kinds of soft bottoms in fresh-water, brackish, and marine habitats (Boxshall and Halsey 2004).

Records of the genus *Nitocra* from Vietnam are still scarce, and only two species, *N. vietnamensis* found in anchialine caves at Ninh Binh province (Tran and Chang 2012), and *N. quangnamensis* found in the hyporheic zone of the Vu Gia-Thu Bon River in Quang Nam Province (Tran et al. 2025b), had been reported from this region. Here we describe a new species, *N. duyxuyenensis* sp. nov., collected from the hyporheic zone in Vu Gia–Thu Bon river (Quang Nam province), Central Vietnam.

## ﻿Materials and methods

Harpacticoid copepods were collected from the hyporheic zone in Vu Gia–Thu Bon river, Quang Nam province, Central Vietnam, by the Karaman-Chappuis method (Karaman 1934; Chappuis 1942). Samples were filtered with a standard zooplankton net with a 50-μm mesh, and subsequently fixed and preserved in 4% formalin. Dissected specimens were mounted in glycerin, and the slides were sealed with transparent nail varnish. Dissected appendages and body ornamentation were examined at 1000 × magnification under an Axio Lab A1 Carl Zeiss compound microscope. All drawings were digitally inked using CORELDRAW v. 19.0®.

Morphological nomenclature follows Huys and Boxshall (1991). Abbreviations used throughout the text are **A1**, antennule; **A2**, antenna; **ae**, aesthetasc; **Exp**, exopod; **Enp**, endopod; **Benp**, baseoendopod; **P1–P6**, first to sixth thoracopod; **Exp(Enp)-1(2, 3)** denotes the proximal (middle, distal) segment of a ramus.

The type material was deposited at the Zoological Collection of Duy Tan University (**ZC-DTU**), Da Nang city, Vietnam.

Two different spellings, *Nitocra* and *Nitokra*, have been widely used [see Gómez in litt., Huys in litt. (Walter and Boxshall 2025)]. The spelling *Nitokra* is regarded as the alternative representation of *Nitocra*, and both are in use (Walter and Boxshall 2025). Here we adopt the spelling *Nitocra* Boeck, 1865 over *Nitokra* Boeck, 1865.

## ﻿Taxonomy

### ﻿Family Ameiridae Boeck, 1865

#### ﻿Genus Nitocra

Taxon classificationAnimaliaHarpacticoidaAmeiridae

Boeck, 1865

7CB1EF7D-FE9C-556C-BB75-61BF763D6FE5

##### Type species.

*Nitocra typica typica* Boeck, 1865.

##### Other species.

*Nitocra affinis* Gurney, 1927; *N. alperi* Yıldız & Karaytuğ, 2024; *N. arctolongus* Shen & Tai, 1973; *N. australis* Soyer, 1975; *N. balli* Rouch, 1972; *N. balnearia* Por, 1964; *N. bdellurae* (Liddell, 1912); *N. bisetosa* Mielke, 1993; *N. blochi* Soyer, 1975; *N. californica* Lang, 1965b; *N. cari* Petkovski, 1954; *N. chelifer* Wilson, C. B., 1932; *N. colombiensis* Fuentes-Reinés & Suárez-Morales, 2014; *N. delaruei* Soyer, 1975; *N. divaricata caspica* Behning, 1936; *N. divaricata divaricata* Chappuis, 1924; *N. dubia* Sars, G. O., 1927; *N. elegans* (Scott, T., 1905); *N. elongata* Marcus, 1968; *N. esbe* Karanovic, Eberhard, Cooper & Guzik, 2015; *N. evergladensis* Bruno & Reid, 2002; *N. fallaciosa baltica* Lang, 1965a; *N. fallaciosa fallaciosa* Klie, 1937; *N. fluviatilis* Galhano, 1968; *N. fragilis fragilis* Sars, G. O., 1905, *N. fragilis paulistana* Jakobi, 1956; *N. galapagoensis* Mielke, 1997; *N. hamata* Bodin, 1970; *N. hibernica bulgarica* (Apostolov, 1976); *N. hibernica hibernica* (Brady, 1880); *N. hibernica hyalina* Jakubisiak, 1929; *N. humphreysi* Karanovic & Pesce, 2002; *N. husmanni* Kunz, 1976; *N. hyperidis* Jakobi, 1956; *N. karanovici* Chullasorn, Kangtia & Klangsin, 2014; *N. kastjanensis* Kornev & Chertoprud, 2008; *N. koreanus* Chang, 2007; *N. lacustris azorica* Kunz, 1983; *N. lacustris colombianus* Reid, 1988; *N. lacustris lacustris* (Schmankevitsch 1875); *N. lacustris pacifica* Yeatman, 1983; *N. lacustris sinoi* Marcus & Por, 1961; *N. laingensis* Fiers, 1986; *N. langi* Karanovic, Eberhard, Cooper & Guzik, 2015; *N. loweae* Yıldız & Karaytuğ, 2024; *N. malaica* Kiefer, 1929; *N. marinae* Jaschnov, 1935; *N. mediterranea jakubisiak* Karanovic, Eberhard, Cooper & Guzik, 2015; *N. mediterranea mediterranea* Brian, 1928; *N. medusaea* Humes, 1953; *N. minor* Willey, 1930; *N. mozambica* Huys, 2021; *N. oligochaeta* Giesbrecht, 1882; *N. phlegraea* Brehm, 1909; *N. phreatica* Bozic, 1964; *N. pietschmanni* Chappuis, 1933; *N. platypus platypus* Daday, 1906; *N. platypus bakeri* Chappuis, 1930; *N. pontica* Boeck, 1865; *N. pori* Karanovic, Eberhard, Cooper & Guzik, 2015; *N. psammophila* Noodt, 1952; *N. pseudospinipes* Yeatman, 1983; *N. puebloviejensis* Fuentes-Reinés, Suárez-Morales & Silva-Briano, 2022; *N. pusilla mediterranea* Brian, 1928; *N. pusilla pusilla* Sars, G. O., 1911; *N. quadriseta* Wells & Rao, 1987; *N. quangnamensis* Tran, Trinh-Dang, Nguyen & Brancelj, 2025; *N. reducta* Schäfer, 1936; *N. reunionensis* Bozic, 1969; *N. rijekana* Petkovski, 1954; *N. salina* (Schmankevitsch, 1875); *N. serdarsaki* Yıldız & Karaytuğ, 2024; *N. sewelli* Gurney, 1927; *N. simplex* Schmeil, 1894; *N. sonmezi* Yıldız & Karaytuğ, 2024; *N. sphaeromata* Bowman, 1988; *N. spinipes orientalis* Sewell, 1924; *N. spinipes spinipes* Boeck, 1865; *N. stygia* Por, 1968; *N. taylori* Gómez, Carrasco & Morales-Serna, 2012; *N. tenuicornis* Brady & Robertson, 1873; *N. typica adriatica* Petkovski, 1954; *N. typica pontica* Jakubisiak, 1938; *N. uenoi* Miura, 1962; *N. vietnamensis* Tran & Chang, 2012; *N. wolterecki* Brehm, 1909; *N. yahiai* (Blanchard & Richard, 1891); *N. yeelirrie* Karanovic, Eberhard, Cooper & Guzik, 2015.

#### ﻿ Nitocra duyxuyenensis sp. nov.

Taxon classificationAnimaliaHarpacticoidaAmeiridae

59F48E8F-1890-5BE7-BACF-643C36019E3F

https://zoobank.org/1D504676-1266-40E7-B0CA-983FE7F6F921

Figs 1, 2, 3, 4, 5, 6, 7, 8, 9

##### Type material.

• Adult female ***holotype*** dissected and mounted on one slide (ZC-DTU-COPEPODA-0014) • adult male ***allotype*** dissected and mounted on one slide (ZC-DTUCOPEPODA-0015) • two female and two male ***paratypes*** preserved in 70% ethanol (ZC-DTU-COPEPODA-0013).

##### Type locality.

Hyporheic zone in Vu Gia – Thu Bon river (15°50'15"N, 108°09'05"E), Quang Nam province, Central Vietnam.

##### Differential diagnosis.

Caudal rami 1.2× as long as wide. Anal operculum with six marginal spinules. Female antennule eight-segmented; male antennule haplocer, eight-segmented. Antenna with allobasis. P1Exp-2 with inner seta; Exp-3 with five elements in all; P1Enp-1 reaching approximately the middle of Exp-2, with inner seta; Enp-2 with three elements. P2–P4Exp-2 with inner seta, Exp-3 with seven setae/spines; P2–P4Enp-1 without, Enp-2 with inner seta, Enp-3 with four, five, and five setae/spines respectively. Female P5Exp with six, Benp with five setae and reaching the middle of Exp; male P5Exp with six, baseoendopods fused medially, each with three setae.

##### Description of the adult female.

**Figure 1.**
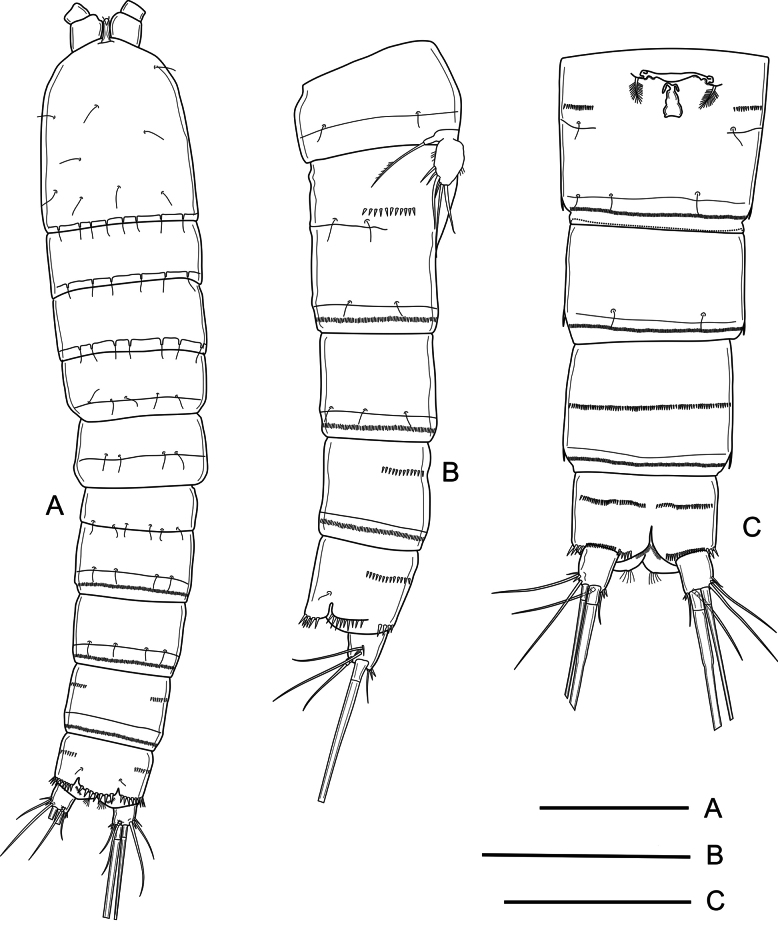
*Nitocra duyxuyenensis* sp. nov. Female, holotype. A. Habitus, dorsal; B. Urosome, lateral; C. Urosome, ventral (P5-bearing somite omitted). Scale bars: 100 µm.

**Figure 2.**
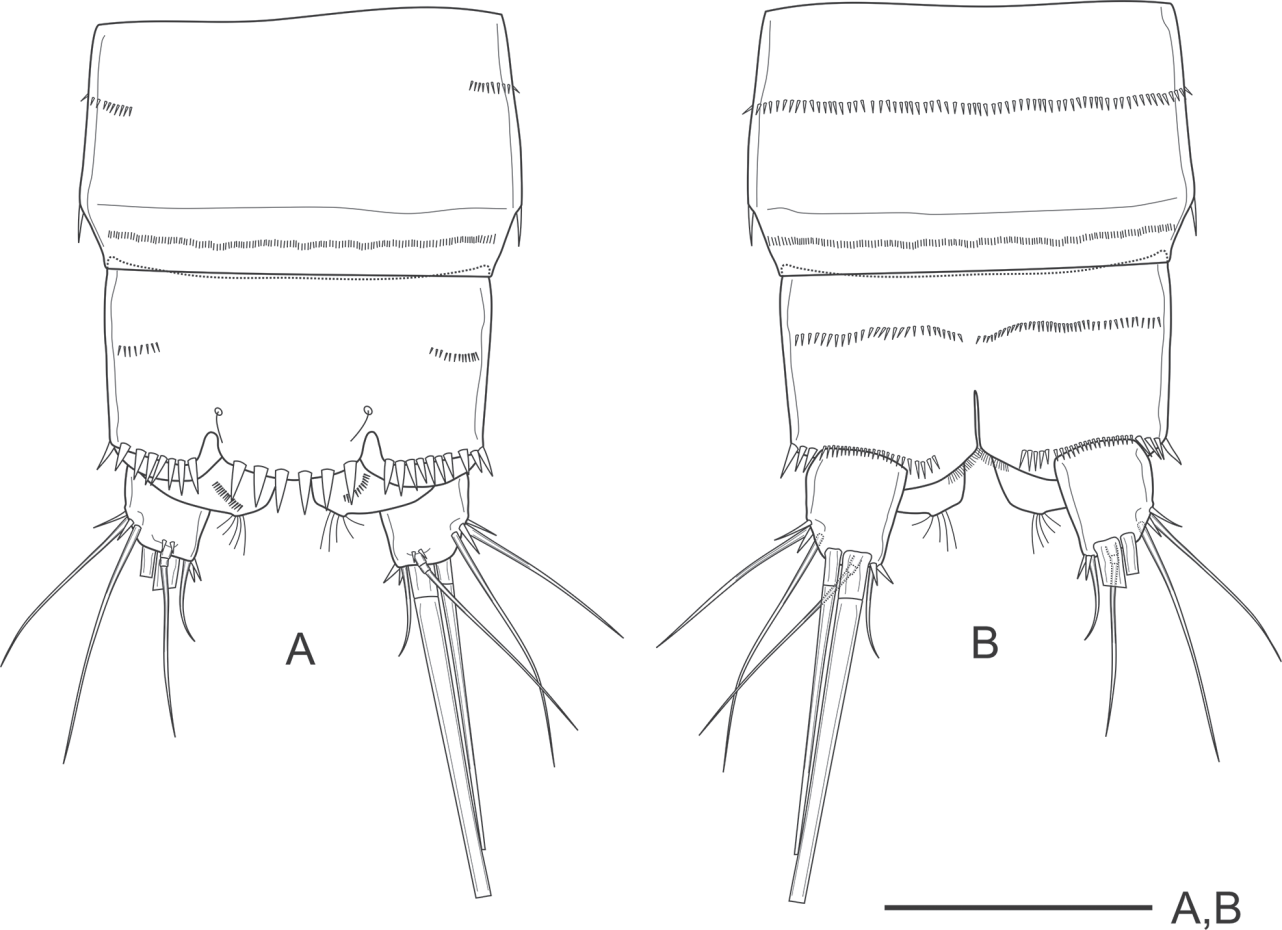
*Nitocra duyxuyenensis* sp. nov. Female, holotype. A. Fifth urosomite, anal somite and caudal rami, dorsal; B. Fifth urosomite, anal somite and caudal rami, ventral. Scale bar: 50 µm.

***Habitus*** (Fig. 1A) semicylindrical; total body length measured from tip of rostrum to posterior margin of caudal rami ranging from 492 to 546 μm (average = 519 μm, *n* = 5; holotype = 546 μm). Nauplius eye not visible. Rostrum (Fig. 1A) linguiform, small, not reaching end of first antennule segment, with pair of tiny sensilla subapically. Prosome four-segmented, comprising cephalothorax with completely fused first pedigerous somite, and three free pedigerous somites; cephalothorax and P2–P4-without surface ornamentation other than sensilla as shown (Fig. 1A). Urosome five-segmented, comprising fifth pedigerous somite, genital double-somite, two free urosomites, and anal somite with caudal rami (Fig. 1A, B); P5-bearing somite without spinular ornamentation, with few posterior sensilla; genital double-somite (Fig. 1A–C) formed by the fusion of genital and third urosomite, with dorsolateral suture indicating original division, completely fused ventrally, proximal half with dorsolateral sensilla close to suture and with short row of lateral spinules as depicted (Fig. 1B), ventrally with medially interrupted row of minute spinules and with sensilla along partial ventral suture, genital field close to anterior margin of first half of somite (Figs 1C, 6B), with large median copulatory pore; fourth urosomite with posterior spinular row ventrally (Fig. 1C); fifth urosomite with medial ventrolateral row of small spinules and with continuous row of small ornaments close to posterior margin, without sensilla (Fig. 2A, B); anal somite (Figs 1A, 2A, B) with median ventrolateral row of spinules, with strong spinules close to caudal rami dorsally (Figs 1A, 2A), and with comparatively smaller ornaments ventrally (Fig. 2B), anal operculum semicircular with six strong marginal spinules and flanked by pair of sensilla (Figs 1A, B, 2A). Caudal rami (Figs 1B, C, 2A, B) short, subquadrate, 1.2× as long as wide, with seven elements each; anterolateral seta I slender, short, ~0.3× as long as caudal ramus; anterolateral seta II and posterolateral seta III smooth, the former slightly shorter than the latter, with short strong spinules at base of seta II; outer apical seta IV and middle apical seta V well-developed, with fracture planes, seta V longest; distal inner accessory seta VI slender, smooth, 0.9 as long as caudal ramus, with several small spinules at its base; dorsal seta VII tri-articulated, ~2.4× as long as caudal ramus, issuing close to posterior margin of ramus.

**Figure 3.**
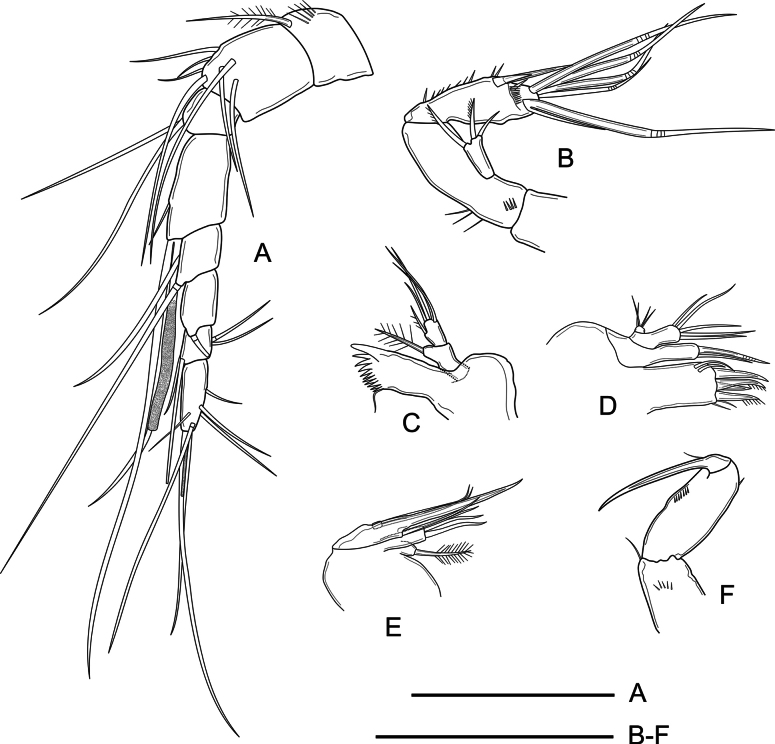
*Nitocra duyxuyenensis* sp. nov. Female holotype. A. Antennule; B. Antenna; C. Mandible; D. Maxillule; E. Maxilla; F. Maxilliped. Scale bars: 50 µm.

***Antennule*** (Fig. 3A) 8-segmented, long and slender; all segments smooth except for first segment with a row of spinules; all setae smooth except for pinnate seta on first segment. Aesthetasc on fourth segment, fused to long seta, reaching well beyond distal end of last segment. Armature formula as follows: 1(1), 2(7), 3(2), 4(2+(1+ae)), 5(2), 6(1), 7(4), 8(5+acrothek).

***Antenna*** (Fig. 3B) relatively short, composed of coxa, allobasis, one-segmented endopod, and one-segmented exopod. Coxa short, unornamented. Allobasis 2.7× as long as wide, with a short row of inner spinules proximally, and several minute proximal spinules close to coxa. Exopod with three distal unequal setae (one bare, two unipinnate). Endopod with inner longitudinal spinular row; lateral armature consisting of one inner spine and one strong inner seta; distally with one smooth seta, four geniculate setae and one distal outer geniculate seta fused basally to slender element.

***Mandible*** (Fig. 3C) with well-developed coxa; gnathobase with ~11 teeth as shown, and single lateral seta. Mandibular palp 2-segmented, first segment (basis) with one strong plumose seta; endopod with one lateral and four smooth apical setae.

***Maxillule*** (Fig. 3D) with well-developed praecoxa; arthrite with two surface setae, and four large robust distal spines. Coxal endite with two smooth setae. Basis with five normal unequal setae distally. Endopod 1-segmented, with four slender smooth setae.

***Maxilla*** (Fig. 3E) without spinular ornamentation on syncoxa, the latter with two endites; proximal endite somewhat bulbous, with one strong plumose seta; distal endite cylindrical, with three subequal setae. Allobasis drawn out into long claw accompanied by a shorter bare seta. Endopod short, one-segmented; with two subequal, smooth setae.

***Maxilliped*** (Fig. 3F) with short and stout syncoxa, the latter 1.3× as long as wide, with short row of posterior spinules, with one small bare seta subapically. Basis ~2.2× as long as wide, with short row of inner spinules and with one outer subdistal spinule. Endopod drawn out into long curved smooth claw accompanied by single short slender seta.

**Figure 4.**
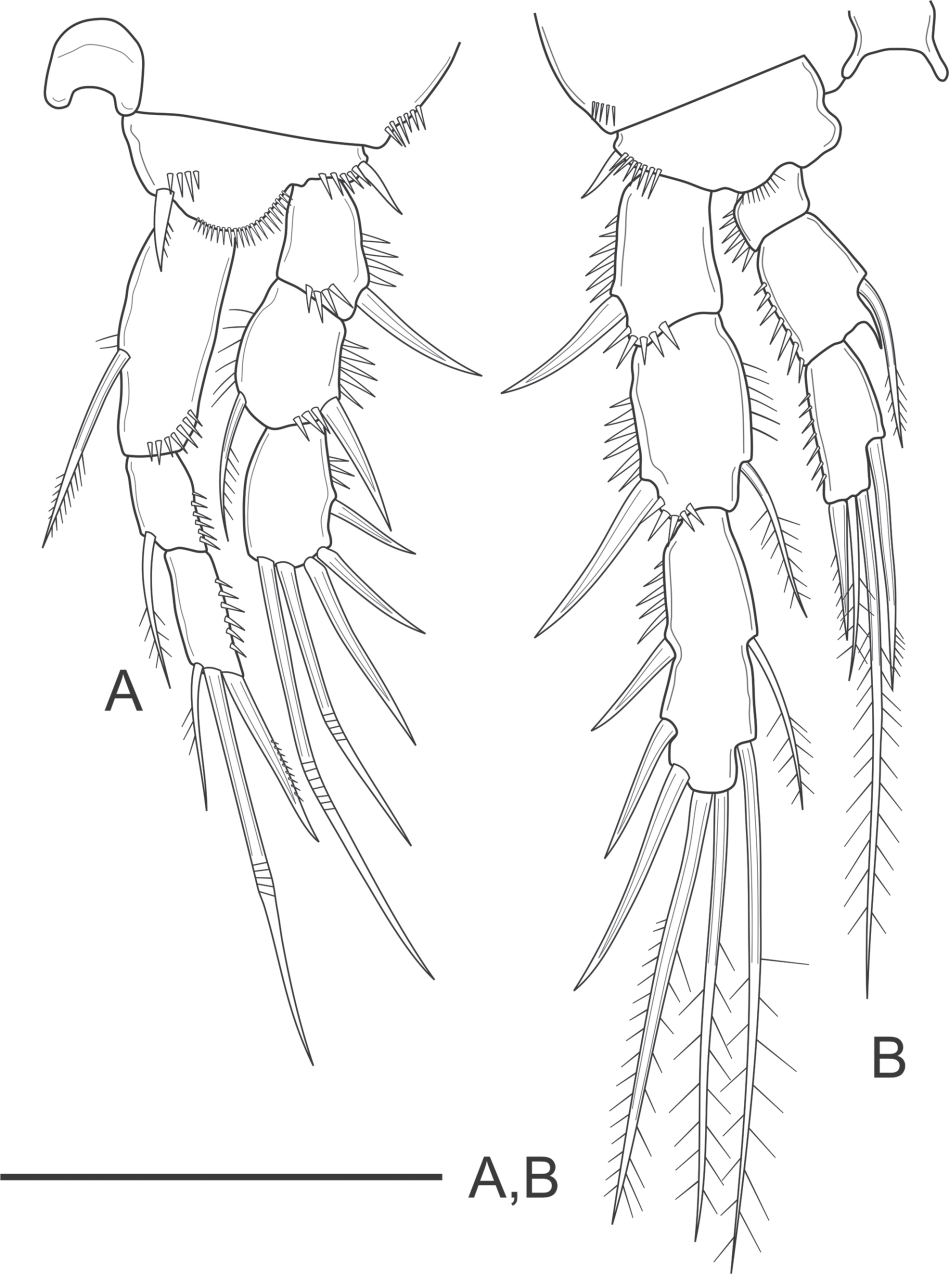
*Nitocra duyxuyenensis* sp. nov. Female, holotype. A. P1; B. P2. Scale bar: 50 µm.

**Figure 5.**
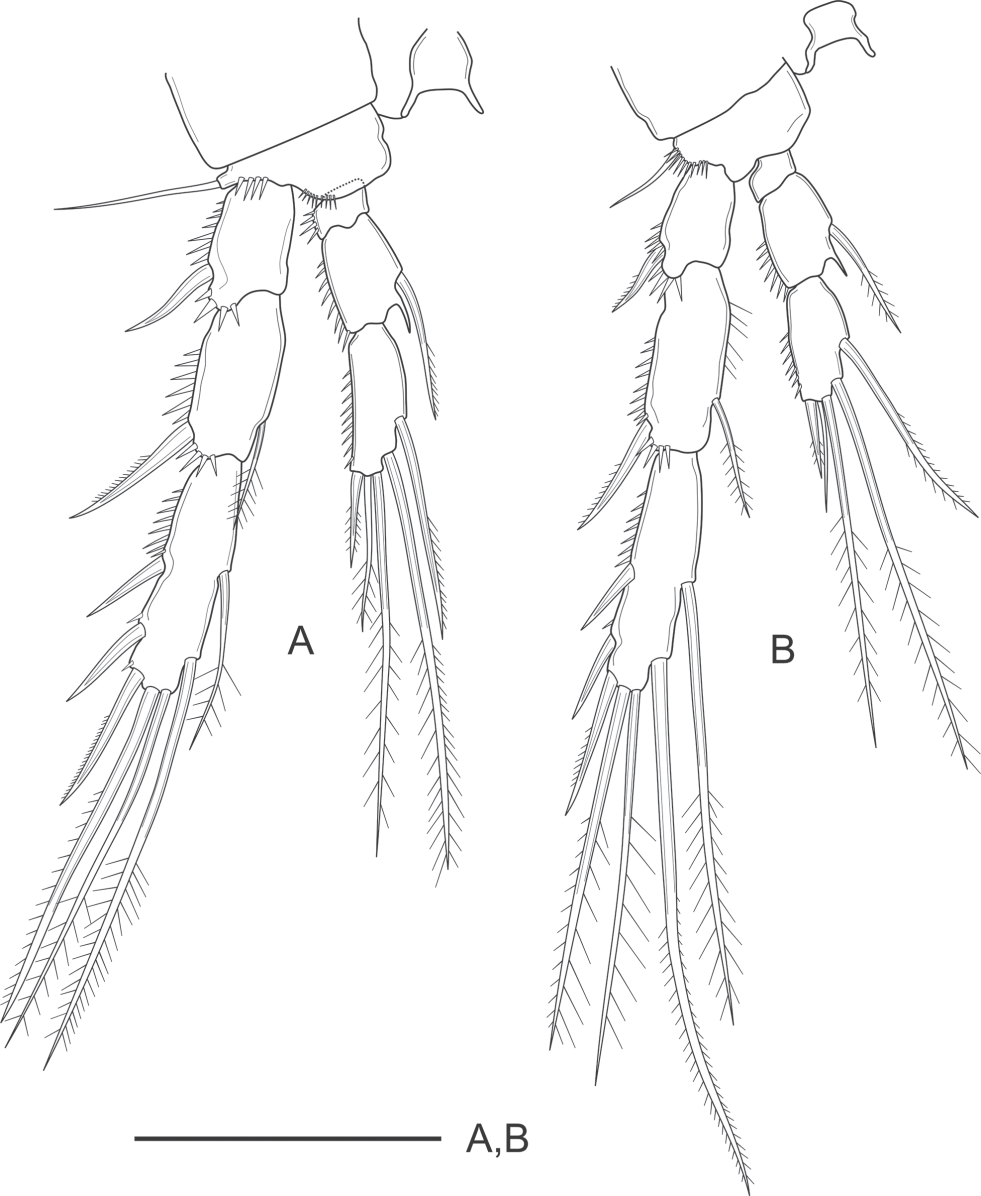
*Nitocra duyxuyenensis* sp. nov. Female holotype. A. P3; B. P4. Scale bar: 50 µm.

***P1–P4*** (Figs 4A, B, 5A, B) with short unornamented intercoxal sclerites, the latter with dorsal tines as shown. Coxa of P1 and P2 with, of P3 and P4 without outer subdistal spinules. Basis of P1 with spinules at the base of inner and outer spine, with additional ornaments between rami; basis of P2–P4 with spinules at the base of outer spiniform (P2) and setiform elements (P3–P4), of P2 and P3 with, of P4 seemingly without spinules close to the base of the endopod. Rami three-segmented. Exopodal segments with outer and subdistal spinules as shown; Exp-1 with outer spine, without inner armature; Exp-2 with outer spine and inner seta; Exp-3 with three outer spines, two distal geniculate setae and without inner armature (P1) or with one distal outer spiniform and one distal inner setiform element and with two inner setae (P2–P4). Endopodal segments with outer ornaments as figured; P1Enp visibly longer than Exp, Enp-1 not reaching beyond Exp-2; Enp-1–2 with inner seta, Enp-3 with three elements; P2–P4Enp shorter than Exp; P2–P3Enp reaching distal tip of Exp-2, P4Enp reaching slightly above the middle of Exp-2; Enp-1 short, wider than long, unarmed; Enp-2 with one inner seta and a projection at the inner distal corner; Enp-3 with one (P2) or two (P3 and P4) inner setae, two distal elements, and one outer spine; length ratio of armature elements on P3Enp-3 (starting from outer most apical spine) 1: 1.9:4.7:5.2:2.9. Armature formula of P1–P4 as in Table 1.

**Table 1.** Armature formula of swimming legs (P1–P4) of *Nitocra duyxuyenensis* sp. nov.

Leg	Basis	Exp			Enp		
		1	2	3	1	2	3
P1	I-I	I-0	I-1	III,2,0	0-1	0-1	I,1,1
P2	0-I	I-0	I-1	III,2,2	0-0	0-1	I,2,1
P3	0-1	I-0	I-1	III,2,2	0-0	0-1	I,2,2
P4	0-1	I-0	I-1	III,2,2	0-0	0-1	I,2,2

**Figure 6.**
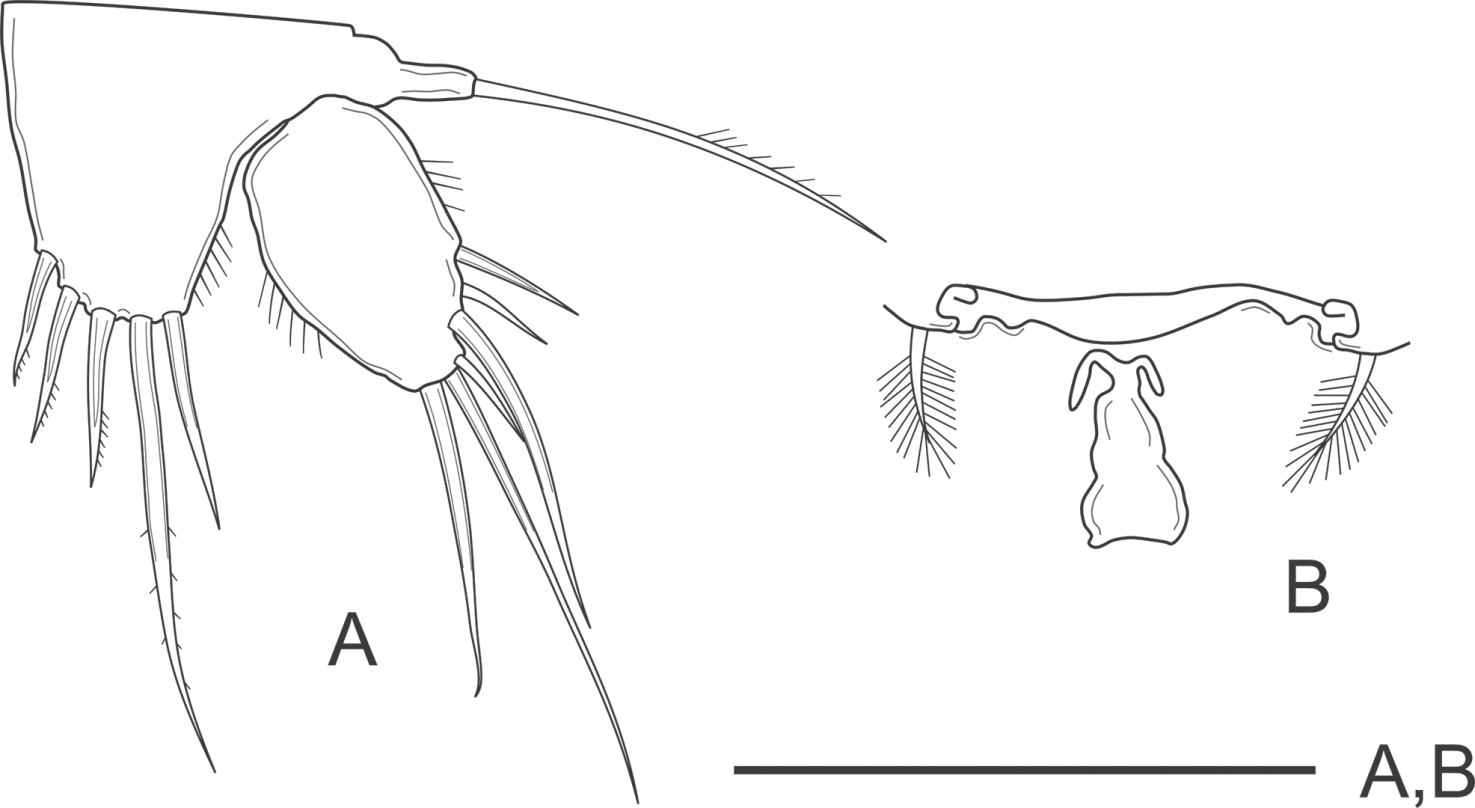
*Nitocra duyxuyenensis* sp. nov. Female holotype. A. P5; B. P6 and genital complex. Scale bar: 50 µm.

***P5*** (Fig. 6A) with exopod and baseoendopod separated. Baseoendopod with outer unipinnate seta; endopodal lobe well-developed, with longitudinal row of outer spinules, with three inner spinulose spines, and two robust distal setae of which distal inner longest. Exopod oval, 1.6× as long as wide, with six unequal smooth setae of which two outer and outer subdistal seta shortest.

***P6*** (Fig. 6B) represented by narrow plate with one single plumose seta on each side.

##### Description of the adult male.

**Figure 7.**
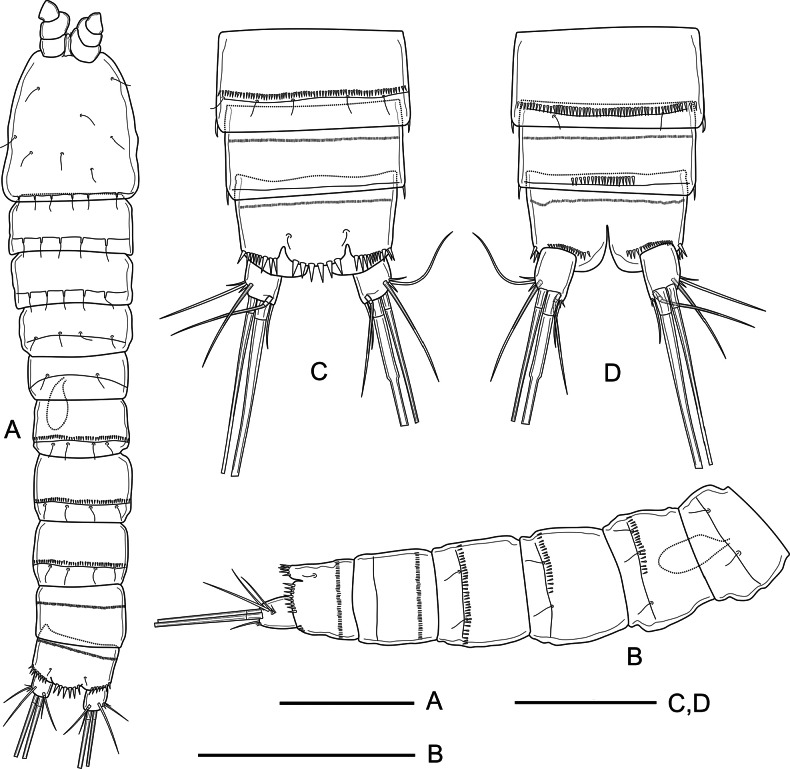
*Nitocra duyxuyenensis* sp. nov. Male allotype. A. Habitus, dorsal; B. Urosome, lateral; C. Fourth and fifth urosomites, and anal somite with caudal rami, dorsal; D. Fourth and fifth urosomites, and anal somite with caudal rami, ventral. Scale bars: 100 µm (A, B), 50 µm (C, D).

Body shape and general appearance as in female (Fig. 7A); total body length measured from tip of rostrum to posterior margin of caudal rami ranging from 476 to 508 μm (average = 492 μm, *n* = 2; holotype = 508 μm). Rostrum, prosomites, anal operculum, and caudal rami as in female (Fig. 7A, C, D). Sexual dimorphism expressed in urosomal segmentation (genital and third urosomite not fused), spinular ornamentation of urosomites, antennule, basis of P1, P5, and P6.

***Genital somite*** (Fig. 7A, B) with continuous posterior dorsolateral spinular row; third urosomite with dorsolateral spinular row interrupted ventrolaterally; fourth and fifth urosomites (Fig. 1A–D) with continuous posterior and median spinular row, respectively, and fifth urosomite with short spinular row ventrally.

**Figure 8.**
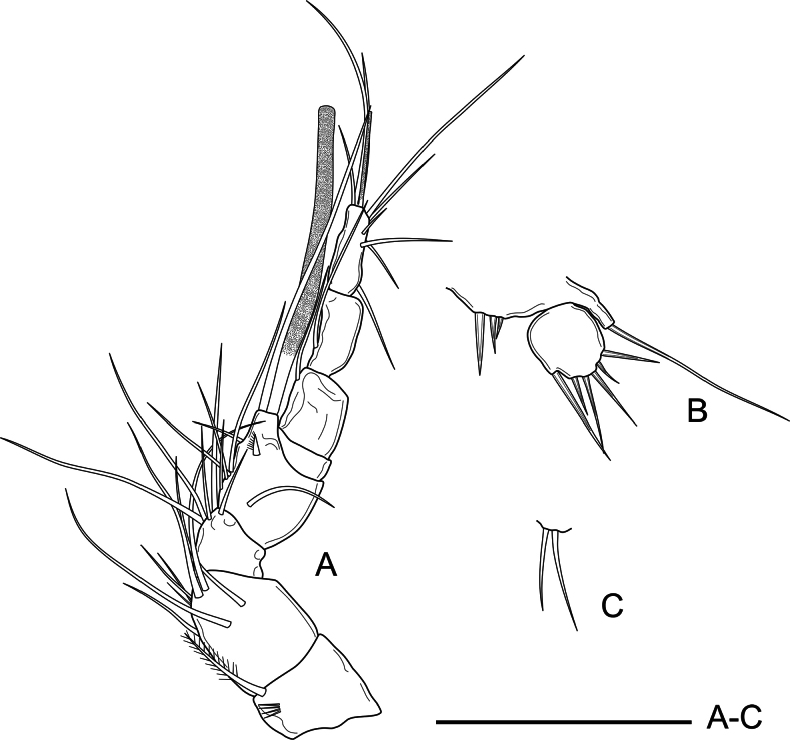
*Nitocra duyxuyenensis* sp. nov. Male allotype. A. A1; B. P5; C. P6. Scale bar: 50 µm.

***Antennule*** (Fig. 8A) haplocer, 8-segmented, with geniculation between sixth and seventh segments; all segments smooth except for first segment with some spinules. All setae smooth except for plumose seta on first segment. Fourth segment with large aesthetasc fused to long seta overreaching last segment, and with one robust short unipinnate spine. Armature formula as follows: 1(1), 2(8), 3(5), 4(6+(1+ae)), 5(0), 6(1), 7(1), 8(7+acrothek).

***Antenna, mandible, maxillule, maxilla, and maxilliped*** (not shown) as in female.

**Figure 9.**
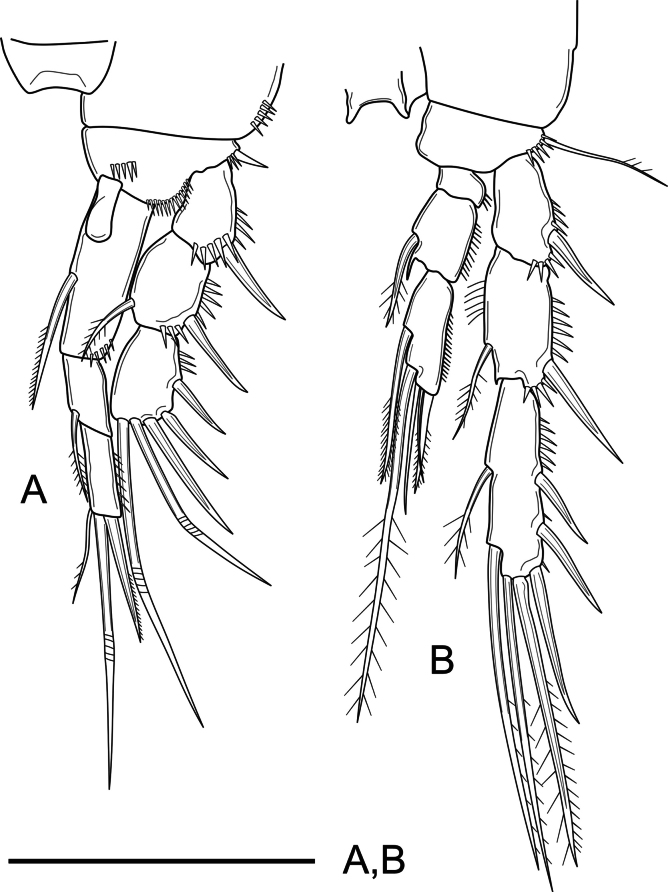
*Nitocra duyxuyenensis* sp. nov. Male allotype. A. P1; B. P3. Scale bar: 50 µm.

***P1*** (Fig. 9A) as in female except for inner spine of basis modified into element with rounded tip; inner margin of Exp-2 and outer margin of Enp-2 naked.

***P2* and P4** (not shown) as in female.

***P3*** (Fig. 9B) as in female except for absence of spinular row on distal margin of basis, and for length ratio of all setae on Enp-3 (1:1.3:1.5:3.6:1.2).

***P5*** baseoendopods (Fig. 8B) fused medially. Baseoendopod with one outer long smooth seta, endopodal lobe weak, with three naked spiniform setae of which outermost shortest, innermost longest. Exopod with six smooth unequal setae; relative length of exopodal setae (from outermost to innermost seta) 1:0.5:1.2:1.3:0.7:1.8.

***P6*** (Fig. 8C) small, with two bare unequal setae, of which inner longer.

##### Variability.

No variability was observed.

## ﻿Discussion

Despite recent advances, the relationships among the species of *Nitocra* are not well understood. Tran and Chang (2012) presented the description of *N. vietnamensis* from a karst area in northern Vietnam, but Gómez et al. (2012) were not aware of this publication and did not include this species in their study in which they split the species of *Nitocra* into three groups based on the combination of the armature formula of P1 Exp2–3.

Group I (*N. sewelli* and *N. platypus bakeri*) was defined by one inner seta and four elements on the P1Exp-2 and Exp-3, respectively. Group II included *N. reducta*, *N. delaruei*, and *N. blochi*. These species lack inner armature on the P1Exp-2, but possess five setae on P1Exp-3. Following Gómez et al. (2012), group III is formed by the remaining species, including *N. vietnamensis*, and was characterized by the presence of one inner, and five elements on the P1Exp-2 and Exp-3, respectively. They also noticed that some species of this group, viz., *N. taylori*, *N. australis*, *N. fragilis*, *N. spinipes*, *N. intermedia*, and *N. husmanni* are unique in the combination of armature elements of the P2–P4Enp-3 and P2–P4Exp-3 (4-5-5, and 7-7-7, respectively) and number of inner setae on the P2–P4Enp-1 (1-1-1). This subgroup is referred here to as the *fragilis* subgroup.

Several papers on the taxonomy and systematics of *Nitocra* were published after 2012. Fuentes-Reinés and Suárez-Morales (2014) described a new subspecies of *N. affinis*, *N. affinis colombiensis*. Chullasorn et al. (2014) described *N. karanovici* from washings of the brown alga *Padina australis*. From the detailed description of the species, it is evident that it belongs to Gómez’s et al. (2012) group III. Karanovic et al. (2015) gave the description of two species, *N. yeelirrie* and *N. esbe*, from Western Australia, attributed here to Gómez’s et al. (2012) group III. Huys (2021) made available Wells’ (1967)*N. minor* Willey, 1930 forma *mozambicœ* n. forma as *N. mozambica*, which is placed here in Gómez’s et al. (2012) group III. Fuentes-Reinés et al. (2022) described *N. puebloviejensis* from northern Colombia and attributed their new species to Gómez’s et al. (2012) group III. *Nitocra puebloviejensis* and *N. taylori*, *N. australis*, *N. fragilis*, *N. spinipes*, *N. intermedia*, and *N. husmanni* share the armature complement of the P2–P4Enp-3 and P2–P4Exp-3 (4-5-5, and 7-7-7, respectively) and number of inner setae on the P2–P4Enp-1 (1-1-1).

In their revision of *N. affinis*, Yıldız and Karaytuğ (2024) described *N. sonmezi* and *N. serdarsaki* from the Turkish coast, *N. alperi* from the Indian Ocean, and *N. loweae* from England; they redescribed *N. affinis* and gave full species rank to all its subspecies. They also noticed that some other species of group III of Gómez et al. (2012), viz., *N. affinis*, *N. colombiensis*, *N. rijekana*, *N. stygia*, *N. hamata*, *N. elegans*, *N. sonmezi*, *N. loweae*, *N. serdarsaki*, and *N. alperi*, constitute another subgroup, the *affinis* subgroup sensu Yıldız and Karaytuğ (2024), defined by the combination of i) the presence of a spinulose, long, spine-like inner middle seta on P4Exp-3, ii) the armature formula of the P2–P4Exp-3 (7-7-8) and P2–P4Enp-3 (4-5-5), iii) the presence of an inner seta on the P2–P4Exp-2, iv) the presence of an inner seta on P2–P4Enp-1 and Enp-2, and v) the elongated P1Enp-1.

Tran et al. (2025b) described *N. quangnamensis* from the hyporheic zone of the Vugia-Thubon River in Vietnam. They correctly attributed *N. quangnamensis* to Gómez et al. (2012) group III, but erroneously presented the species as part of Yıldız and Karaytuğ’s (2024)*affinis* subgroup in their table 3 [see Tran et al. (2025b: 57)].

Based on the armature complement of Exp-2 and Exp-3 of P1, *Nitocra duyxuyenensis* sp. nov. is placed here into Gómez’s et al. (2012) group III but it cannot be included in the *fragilis* (the new species lacks inner armature on P2–P4Enp-1), or *affinis* subgroup (the new species possesses seven instead of eight elements on P4Exp-3, lacks the strong middle inner spiniform element on P4Exp-3 and an inner seta on P2–P4Enp-1, and possesses a comparatively shorter P1Enp-1), and the *lacustris* subgroup is proposed herein for *N. balli*, *N. duyxuyenensis* sp. nov., *N. evergladensis*, *N. lacustris pacifica*, and *N. pseudospinipes*. The *lacustris* subgroup is defined by the armature formula of P2–P4Enp-3 (4-5-5), P2–P4Exp-3 (7-7-7) and by the lack of inner armature on the P2–P4Enp-1 in females. A comparative table with the taxa attributed so far to the *affinis*, *fragilis*, and *lacustris* subgroups is presented below (see Tables 2–4). The key characters and character states selected for comparison include i) the relative length of P1Enp-1, ii) armature formula of P2–P4Enp-1, iii) armature formula of P2–P4Enp-3, iv) armature formula of P2–P4Exp-3, v) the number of inner setae on P4Exp-3, vi) the setation of P5Exp/baseoendopod in both females and males, vii) the segmentation of A2Enp, and viii) the relative length of the female P5 baseoendopod.

**Table 2.** Differentiating characters of the species of the *affinis* subgroup. uk = unknown.

Species	P1Enp-1	P5 female Exp:Benp	P5 male Exp:Benp	Female P5Benp	A2	Female P5Exp	Rostral projection	Inner seta of P1Enp-1	P1Enp-3	P5 Benp	Maxilliped syncoxa
	relative length	# setae	# setae	relative length	allobasis/ basis	l/w		position, ornamentation, and relative length	# setae	Hyaline area	# setae
N. affinis	beyond Exp-3	6:5	6:4–5	1/2 Exp	allobasis	1.8	with	2/3 Enp-1; bipinnate; > Enp	3	without	1
N. californica	beyond Exp-3	6:5	6:4	2/3 Exp	uk	2–2.2	uk	2/3 Enp-1; unipinnate; ~Enp	3	without	uk
N. colombiensis	beyond Exp-3	6:5	6:3	1/2 Exp	basis	1.6	with	1/4 Enp-1; bipinnate; > Enp	3	without	1
N. rijekana	uk	6:5	6:5	1/2 Exp	uk	uk	uk	uk	uk	without	uk
N. stygia	beyond Exp-3	6:5	6:4	1/2 Exp	uk	1.3	uk	1/4 Enp-1; bipinnate; ~Enp	2	with, large	uk
N. hamata	beyond Exp-3	6:5	uk	1/4 Exp	basis	4.4	uk	1/2 Enp-1; unipinnate; ~ Enp	3	without	2
N. elegans	beyond Exp-3	6:5	6:4	1/3 Exp	basis	1.9	uk	2/3 Enp-1; bipinnate; > Enp	3	without	1
N. sonmezi	beyond Exp-3	6:5	uk	1/2 Exp	basis	1.7	Without	2/3 Enp-1; plumose; ~1/2 Enp-3	3	without	1
N. loweae	~Exp-3	6:5	6:5	1/3 Exp	basis	1.5	Without	1/4 Enp-1; pinnate; > Enp	3	without	1
N. serdarsaki	beyond Exp-3	uk	6:4	uk	allobasis	uk	Without	2/3 Enp-1; unipinnate; ~Enp	3	without	1
N. alperi	beyond Exp-3	6:5	uk	1/2 Exp	basis	1.7	with	2/3 Enp-1; unipinnate; ~Enp	3	without	1

**Table 3.** Differentiating characters of the species of the *lacustris* subgroup. uk = unknown.

Species	P1Enp-1	P5 female Exp:Benp	P5 male Exp:Benp	Female P5Benp:Exp	A2	A2Exp-2	Female P6	Inner seta Enp-1 P1	Anal operculum	CR
	relative length	# setae	# setae	relative length	allobasis/ basis	# setae	# setae	position, ornamentation, and relative length	ornamentation	l/w
N. duyxuyenensis sp. nov.	~Exp-2	6:5	6:3	1/2 Exp	allobasis	3	1	1/2 Enp-1; bipinnate; ~Enp-2	6 strong spinules	~1
N. karanovici	2/3 Exp-3	5:5	6:3	1/3 Exp	basis	3	1	3/4 Enp-1; plumose; ~Enp-3	11 strong spinules	~1
N. evergladensis	~Exp-2	6:5	7:4	2/3 Exp	basis	3	uk	2/3 Enp-1; smooth; ~1/2 Enp-3	7 strong spinules	~1
N. balli	1/2 Exp-3	5:4	5–6:2	1/3 Exp	basis	3	2	2/3 Enp-1; smooth; ~Enp-2	9–11 spinules	~0.7
N. pseudospinipes	1/2 Exp-2	6:3–4	5–6:3	1/3 Exp	basis	3	uk	1/2 Enp-1; smooth; ~1/2 Enp-3	8 small spinules	0.4
N. lacustris pacifica	1/2 Exp-2	6:5	6:2	1/2 Exp	basis	uk	1	uk	2–4 large spines	1.5
N. fallaciosa baltica	~Exp-2	5:5	6:4	uk	uk	uk	2	2/3 Enp-1; smooth; > Enp-3	~10 spinules	1.3
N. minor	beyond Exp-3	5:5	5:2	uk	uk	3	uk	2/3 Enp-1; smooth; ~1/2 Enp-2	11 spinules	~0.7
N. phreatica	~Exp-3	5:3	4:2	uk	uk	3	uk	2/3 Enp-1; smooth; ~Enp-2	12 spinules	~1
N. psammophila	1/2 Exp-3	6:5	6:3	1/2 Exp	uk	3	uk	1/2 Enp-1; smooth; ~Enp-3	~6 spinules	1.2
N. uenoi	~Exp-2	5:4	uk	1/2 Exp	basis	2	1	3/4 Enp-1; smooth; ~Enp-2	11 spinules	1.2

**Table 4.** Differentiating characters of the species of the *fragilis* subgroup. uk = unknown.

Species	P1Enp-1	Female P5Exp:Benp	Male P5Exp:Benp	Female P5Benp	A2	Female P6	Inner seta P1Enp-1	P1Enp-3	Anal operculum	CR
	relative length	# setae	# setae	relative length	allobasis/ basis	# setae	position, ornamentation, and relative length	# setae	ornamentation	l/w
N. australis	~Exp--3	5:5	6:4	1/2 Exp	basis	3	2/3 Enp-1; plumose; ~Enp-3	3	~22 large spinules	~0.7
N. fragilis	1/2 Exp-3	5:5	6:3	1/2 Exp	basis	uk	2/3 Enp-1; smooth; ~Enp-3	2	~12 spinules	~0.7
N. intermedia	2/3 Exp-3	5:5	6:3	1/2 Exp	allobasis	uk	2/3 Enp-1; bipinnate; ~Enp-3	3	~7–8 spinules	uk
N. husmanni	~Exp-2	6:5	6:3	~2/3 Exp	uk	uk	2/3 Enp-1; bipinnate; ~Enp-2	3	~7 spinules	~1
N. pietschmani	1/2 Exp-2	6:5	6:3	2/3 Exp	allobasis	uk	2/3 Enp-1; plumose; ~Enp-3	3	~4–6 spinules	~1
N. laingensis	uk	5:4	uk	1/2 Exp	uk	2	uk	uk	6 large spinules	~1
N. koreanus	~Exp-2	6:5	6:3	1/3 Exp	allobasis	uk	2/3 Enp-1; bipinnate; ~Enp-3	3	4–5 spinules	~1
N. taylori	2/3 Exp-2	6:5	6:2	1/3 Exp	allobasis	2	2/3 Enp-1; bipinnate; > Enp-3	3	3 strong spinules	1.2
N. vietnamensis	1/2 Exp-3	6:5	6:3	1/2 Exp	allobasis	2	2/3 Enp-1; bipinnate; ~Enp-3	3	5–6 strong spinules	~1
N. puebloviejensis	2/3 Exp-3	5:5	6:4	1/2 Exp	basis	1	1/2 Enp-1; bipinnate; ~Enp-3	3	5–6 spinules	~1
N. spinipes	1/2 Exp-3	5:5	6:5	1/2 Exp	basis	3	2/3 Enp-1; bipinnate; > Enp-3	3	8–12 spinules	~1
N. sphaeromata	beyond Exp-3	5:5	5:4	1/2 Exp	basis	uk	2/3 Enp-1; unipinnate; ~Enp-2	3	With row spinules	~1
N. reunionensis	~Exp-2	5:4	6:2	1/2 Exp	uk	1	2/3 Enp-1; bipinnate; ~Enp-2	3	~7 large spinules	~1
N. humphreysi	~Exp-2	4–5:4	5–6:3	1/2 Exp	basis	1	2/3 Enp-1; bipinnate; ~Enp-3	3	~5 strong spines	~1
N. quangnamensis	2/3 Exp-3	6:4	5–6:3	1/2 Exp	basis	1	1/2 Enp-1; bipinnate; ~Enp-2	3	6 strong spines	~1

*Nitocra duyxuyenensis* sp. nov. is similar to *N. evergladensis* but they differ in i) the anal operculum (furnished with six spines in both sexes of the new species, but with seven spines in *N. evergladensis*); ii) the relative length of the female P5 baseoendopod (reaching the middle of Exp in *N. duyxuyenensis* sp. nov., but comparatively longer in *N. evergladensis*); iii) A2 with allobasis in the new species, but with basis in *N. evergladensis*; iv) the setal complement of the male P5Exp/Benp (6/3 in *N. duyxuyenensis* sp. nov., but 7/4 in *N. evergladensis*); v) the number of setae on the inner margin of the male P2–P4Enp-1 (0-0-0 in the new species, but 1-1-1 in *N. evergladensis*); vi) armature complement of P3Enp-3 (with four setae and one spine in the new species, but with three setae and one spine in *N. evergladensis*).

Several species (see Table 5) do not fit in any of the subgroups above. Some of them, whose grouping is referred below to as the *mediterranea* subgroup (*N. mediterranea jakubisiak*, *N. mediterranea mediterranea*, *N. typica adriatica*, *N. elongata*, *N. langi*, *N. fluviatilis*) are similar to the *affinis* and *fragilis* subgroups in the presence of an inner seta on P2–P4Enp-1, but display a reduced number of setae and spines on P2–P4Enp-3 and Exp-3. Similarly, some other species (*N. kastjanensis*, *N. quadriseta*, *N. cari*, *N. arctolongus*, *N. yeelirrie*, *N. bisetosa*, *N. esbe*) are similar to the *lacustris* subgroup in that they lack inner armature on P2–P4Enp-1, but also display a reduced number of inner setae on P2–P4Enp-3 and Exp-3. The phylogenetic relationships among the species of *Nitocra* are far from resolved and an extensive analysis with more (micro)characters might be useful to better understand their relationships.

**Table 5.** Differentiating characters of the species of the *mediterranea* subgroup. uk = unknown.

Species	P1Enp-1	P2–P4Enp-1	P2–P4Enp-3	P2–P4Exp-3	P4Exp-3	Female P5Exp:Benp	Female P5Benp	Male P5Exp:Benp	A2
	relative length	# inner setae	# setae	# setae	# inner setae	# setae	relative length	# setae	allobasis/ basis
N. mediterranea jakubisiak	uk	1-1-1	5-4-5	6-6-6	1	6:5	uk	uk	uk
N. mediterranea mediterranea	~Exp-3	1-1-1	4-4-5	6-6-6	1	6:5	1/2 Exp	5:4	basis
N. typica adriatica	2/3 Exp-3	1-1-1	4-4-4	6-6-6	1	6:5	1/3 Exp	6:4	uk
N. elongata	~Exp-3	1-1-1	4-4-4	6-6-7	2	5:5	1/2 Exp	6:4	basis
N. langi	uk	1-1-1	4-4-4	4-6-7	2	5:5	uk	uk	uk
N. fluviatilis	~Exp-2	1-1-1	3-3-3	5-5-7	2	6:4	1/2 Exp	6:3	basis
N. kastjanensis	~Exp-2	0-0-0	5-5-5	7-7-7	2	5:5	uk	6:3	basis
N. quadriseta	~Exp-3	0-0-0	4-4-4	7-7-7	2	5:5	uk	6:4	basis
N. cari	~Exp-3	0-0-0	3-5-5	7-7-8	3	6:4	uk	5:2	uk
N. arctolongus	~Exp-2	0-0-0	3-4-5	7-7-7	2	5:5	1/3 Exp	6:2	allobasis
N. yeelirrie	1/2 Exp-2	0-0-0	3-4-4	7-7-7	2	6:4	1/3 Exp	6:2	allobasis
N. bisetosa	~Exp-3	0-0-0	3-4-4	7-7-6	1	5:2	1/3 Exp	6:2	basis
N. esbe	~Exp-2	0-0-0	3-4-4	6-6-7	2	7:4	1/3 Exp	6:2	allobasis

The dense river network in Central Vietnam, originating from the Annamite Range, characterized by steep terrain and widespread sandy sediments, has created particularly favorable geomorphological and hydrological conditions for the formation and development of the hyporheic zone. This is an intermediate zone between surface water and groundwater, where metabolic processes and micro-flows take place, providing a stable habitat for many species of benthic organisms and plankton. To date, only four species of copepods (*Parastenocaris sontraensis* Tran, Trinh-Dang & Brancelj, 2021; *P. vugiaensis* Tran, Trinh-Dang & Brancelj, 2021; *Nitocraquangnamensis, Phyllognathopusvietnamensis* Tran, Nguyen & Brancelj, 2025) have been discovered from the hyporheic region in Vietnam, suggesting that the copepod biodiversity in this ecosystem remains considerably underestimated (Tran et al. 2025b).

Identification keys to the species groups and species of each subgroup of *Nitocra* are given below. The identification of the species of *Nitocra* is difficult mainly because of poor original descriptions of some species. The user is suggested to also check the original descriptions in case of doubt.

### ﻿Key to the groups and subgroups of *Nitocra*

**Table:** 

1	P1Exp-3 with four spines/setae	**group I**
–	P1Exp-3 with five spines/setae	**2**
2	P1Exp-2 without inner seta	**group II**
–	P1Exp-2 with inner seta	**3 (group III)**
3	P2–P4Enp-3 with 4:5:5 spines/setae	**4**
–	Number of spines/setae on P2–P4Enp-3 different	***mediterranea* subgroup**
4	P2–P4Enp-1 without inner seta	***lacustris* subgroup**
–	P2–P4Enp-1 with one inner seta	**5**
5	P2–P4Exp-3 with 7:7:8 spines/setae	***affinis* subgroup**
–	P2–P4Exp-3 with 7:7:7 spines/setae	***fragilis* subgroup**

### ﻿Key to the species of the *affinis* subgroup

**Table:** 

1	Inner middle seta of P4Exp-3 not stronger, but longer than the other setae	***N. rijekana***
–	Inner middle seta of P4 exopod-3 stronger and longer than the other setae, and spinulose	**2**
2	P5Exp l/w ratio ≥ 4; syncoxa of maxilliped with 2 setae	***N. hamata***
–	P5Exp l/w ratio < 4; syncoxa of maxilliped with 1 seta	**3**
3	Rostrum with rostral projection	**4**
–	Rostrum without rostral projection	**6**
4	A2 with allobasis	***N. affinis***
–	A2 with basis	**5**
5	Inner seta of P1Enp-1 bipinnate, inserted at distal 1/4 of segment, reaching beyond Enp-3; all setae of EnpP2–P4 smooth	***N. colombiensis***
–	Inner seta of P1Enp-1 bipinnate, inserted at 2/3 of segment, reaching approximately the tip of Enp-3; all setae of EnpP2–P4 pinnate	***N. alperi***
6	P1Enp-3 with 2 setae; P5 baseoendopod with large hyaline field	***N. stygia***
–	P1Enp-3 with 3 setae; P5 baseoendopod without hyaline field	**7**
7	P1Enp-1 barely reaching tip of Exp-3; inner seta of P1Enp-1 inserted at distal 1/4 of segment	***N. loweae***
–	P1Enp-1 reaching beyond tip of Exp-3; inner seta of P1Enp-1 inserted at distal 2/3 of segment	**8**
8	A2 with allobasis	***N. serdarsaki***
–	A2 with basis	**9**
9	Inner seta of P1Enp-1 bipinnate; female P5 baseoendopod reaching proximal 1/3 of Exp	***N. elegans***
–	Inner seta of P1Enp-1 strongly unipinnate; female P5 baseoendopod reaching beyond 1/2 of Exp	**10**
10	L/w ratio of female P5Exp ~1.7, baseoendopod reaching the middle of Exp, with innermost seta shortest	***N. sonmezi***
–	L/w ratio of female P5Exp ~2–2.2, baseoendopod not reaching proximal 2/3 of Exp, with outermost seta shortest	***N. californica***

### ﻿Key to the species of the *lacustris* subgroup

**Table:** 

1	Female P5Exp with 5 setae	**2**
–	Female P5Exp with 6 setae	**7**
2	P1Enp-1 reaching beyond Exp-3	***N. minor***
–	P1Enp-1 at most as long as Exp-3	**3**
3	A2Exp one-segmented with 2 setae	***N. uenoi***
–	A2Exp one-segmented with 3 setae	**4**
4	Female P5, baseoendopod with 3 setae	***N. phreatica***
–	Female P5, baseoendopod with 4 or 5 setae	**5**
5	Inner seta of P1Enp-1 inserted at ¾ of segment; female and male P5Exp and baseoendopod with 5 and 5, and 6 and 3 setae, respectively	***N. karanovici***
–	Inner seta of P1Enp-1 inserted at 2/3 of segment; female and male P5Exp and baseoendopod with different number of setae	**6**
6	Female and male P5Exp and baseoendopod with 5 and 4, and 5 or 6 and 3 setae, respectively; P1Enp-1 reaching the middle of Exp-3	***N. balli***
–	Female and male P5 Exo and baseoendopod with 5 and 5, and 6 and 4 setae, respectively; P1Enp-1 reaching the tip of Exp-2	***N. fallaciosa baltica***
7	P1Enp-1 reaching beyond Exp-2	***N. psammophila***
–	P1Enp-1 not reaching beyond Exp-2	**8**
8	A2 with allobasis	***N. duyxuyenensis* sp. nov.**
–	A2 with basis	**9**
9	Inner seta of P1Enp-1 inserted at 1/2 of segment; female P5 baseoendopod with 3 or 4 setae	***N. pseudospinipes***
–	Inner seta of P1Enp-1 inserted at 2/3 of segment; female P5 baseoendopod with 5 setae	**10**
10	Male P5Exp and baseoendopod with 7 and 4 setae, respectively; P1Enp-1 reaching Exp-2	***N. evergladensis***
–	Male P5Exp and baseoendopod with 6 and 2 setae, respectively; P1Enp-1 reaching ½ of Exp-2	***N. lacustris pacifica***

### ﻿Key to the species of the *fragilis* subgroup

**Table:** 

1	Female P5Exp with 4–5 setae	**2**
–	Female P5Exp with 6 setae	**10**
2	Female P5 baseoendopod with 4 setae	**3**
–	Female P5 baseoendopod with 5 setae	**5**
3	Female P6 with 2 setae	***N. laingensis***
–	Female P6 with 1 seta	**4**
4	Male P5Exp and baseoendopod with 6 and 2 setae, respectively	***N. reunionensis***
–	Male P5Exp and baseoendopod with 5–6 and 3 setae, respectively	***N. humphreysi***
5	A2 with allobasis	***N. intermedia***
–	A2 with basis	**6**
6	P1Enp-3 with 2 setae	***N. fragilis***
–	P1Enp-3 with 3 setae	**7**
7	P1Enp-1 reaching beyond Exp-3	***N. sphaeromata***
–	P1Enp-1 not reaching beyond Exp-3	**8**
8	Female P6 with 1 short seta; inner seta of P1Enp-1 inserted midway inner margin of segment	***N. puebloviejensis***
–	Female P6 with 3 setae; inner seta of P1Enp-1 inserted at distal 2/3 of segment	**9**
9	P1Enp-1 reaching approx. tip of Exp-3; male P5Exp and baseoendopod with 6 and 4 setae, respectively	***N. australis***
–	P1Enp-1 reaching the middle of Exp-3: male P5Exp and baseoendopod with 6 and 5 setae, respectively	***N. spinipes***
10	Female P5 baseoendopod with 4 setae	***N. quangnamensis***
–	Female P5 baseoendopod with 5 setae	**11**
11	Male P5 baseoendopod with 2 setae	***N. taylori***
–	Male P5 baseoendopod with 3 setae	**12**
12	P3Exp-3 with middle outer seta not reaching beyond tip of segment; inner seta of P1Enp-1 reaching approx. tip of Enp-2	***N. husmanni***
–	P3Exp-3 with middle outer seta reaching beyond tip of segment; inner seta of P1Enp-1 reaching approx. tip of Enp-3	**13**
13	Female P5Exp rounded, baseoendopod reaching ~2/3 of Exp	***N. pietschmanni***
–	Female P5ExpP5 ellipsoidal, baseoendopod reaching 1/2 or 1/3 of Exp	**14**
14	P1Enp-1 reaching tip of Exp-2; female P5 baseoendopod reaching ~2/3 of Exp, all setae pinnate; male P5Exp with fifth seta from inner to outer shortest	***N. koreanus***
–	P1Enp-1 reaching 1/2 of Exp-3; female P5 baseoendopod reaching ~1/2 of Exp, all setae smooth; male P5Exp with second seta from inner to outer shortest	***N. vietnamensis***

### ﻿Key to the species of the *mediterranea* subgroup

**Table:** 

1	P2–P4Enp-1 with 1:1:1 inner seta, respectively	**2**
–	P2–P4Enp-1 without inner seta	**7**
2	P4Exp-3 with 1 inner seta	**3**
–	P4Exp-3 with 2 inner setae	**5**
3	P2Enp-3 with 5 setae	***N. mediterranea jakubisiak***
–	P2Enp-3 with 4 setae	**4**
4	P4Enp-3 with 5 setae; male P5Exp and baseoendopod with 5 and 4 setae, respectively	***N. mediterranea mediterranea***
–	P4Enp-3 with 4 setae; male P5Exp and baseoendopod with 6 and 4 setae, respectively	***N. typica adriatica***
5	P2–P4Enp-3 with 3:3:3 setae/spines, respectively; female P5Exp and baseoendopod with 6 and 4 setae, respectively	***N. fluviatilis***
–	P2–P4Enp-3 with 4:4:4 setae/spines, respectively; female P5Exp and baseoendopod with 5 setae each	**6**
6	P2–P4Exp-3 with 6:6:7 setae/spines, respectively	***N. elongata***
–	P2–P4Exp-3 with 4:6:7 setae/spines, respectively	***N. langi***
7	P4Exp-3 with 1 or 3 inner setae	**8**
–	P4Exp-3 with 2 inner setae	**9**
8	P2–P4Enp-3 with 3:5:5 setae/spines, respectively; P4Exp 3 with 8 setae/spines; female and male P5Exp and baseoendopod with 6 and 4, and 5 and 2 setae, respectively	***N. cari***
–	P2–P4Enp-3 with 3:4:4 setae/spines, respectively; P4Exp-3 with 6 setae/spines; female and male P5Exp and baseoendopod with 5 and 2, and 6 and 2 setae, respectively	***N. bisetosa***
9	A2 with allobasis	**10**
–	A2 with basis	**12**
10	P2–P4Exp-3 with 6:6:7 setae/spines, respectively	***N. esbe***
–	P2–P4Exp-3 with 7:7:7 setae/spines, respectively	**11**
11	P4Enp-3 with 5 setae/spines; female P5Exp and baseoendopod with 5 setae each	***N. arctolongus***
–	P4Enp-3 with 4 setae/spines; female P5Exp and baseoendopod with 6 and 4 setae, respectively	***N. yeelirrie***
12	P2–P4Enp-3 with 5:5:5 setae/spines, respectively; P1Enp-1 reaching tip of Exp-2; female P5Exp and baseoendopod with 6 and 3 setae, respectively	***N. kastjanensis***
–	P2–P4Enp-3 with 4:4:4 setae/spines, respectively; P1Enp-1 reaching tip of Exp-3; female P5Exp and baseoendopod with 6 and 4 setae, respectively	***N. quadriseta***

## Supplementary Material

XML Treatment for Nitocra

XML Treatment for Nitocra duyxuyenensis

## ﻿Additional information

### Use of AI

No use of AI was reported.

### Author ORCIDs

Ngoc-Son Tran https://orcid.org/0000-0003-2387-5822

Thi-Phuong Pham https://orcid.org/0009-0003-4203-6183

Khanh Chuyen Phungn https://orcid.org/0009-0008-3615-3216

Samuel Gómez https://orcid.org/0000-0002-8597-8846

### Data availability

All of the data that support the findings of this study are available in the main text.
